# Adverse events in patients in a Brazilian intensive care according to the readmission

**DOI:** 10.1186/cc14605

**Published:** 2015-03-16

**Authors:** GM Plantier, CE Bosso, AC Correa, BN Azevedo, JS Alves, CD Monteiro, SV Ferreira, GE Valerio, JV Moraes, V Raso

**Affiliations:** 1 Instituto do Coração de Presidente Prudente, Brazil; 2 Santa Casa de Presidente Prudente, Brazil; 3 Faculdade de Medicina - UNOESTE, Presidente Prudente, Brazil

## Introduction

Security policies for the patient are of interest to any health professional. The rates of adverse events in hospitals reach values ranging between 3.7 and 16.6%, being the highest range (40 to 70%) considered preventable or avoidable [1]. The objective of this study is to assess the prevalence and types of adverse effects in intensive care patients according to readmission.

## Methods

During 4 years the data from 2,582 patients admitted to the coronary ICU (CICU) were analyzed in the coronary care unit of the city of Presidente Prudente, Brazil. We analyzed the rate of readmissions, length of stay and the mainly detected adverse events. We considered significant *P *< 0.05 two-tailed and confidence intervals at 95% (CI).

## Results

The readmission rate was 15% (*n *= 392), particularly among males (55% (*n *= 214)). A 14.3% adverse event rate was observed among the readmitted patients (9.5% among those who were not readmitted). The readmitted patients were older (median (md): 68.0 (95% CI: 65.7 to 67.3) vs. md: 71.0 (95% CI: 67.1 to 71.3); *P *< 0.05) and remained hospitalized for a longer period of time (md: 5.0 (95% CI: 13.2 to 20.7) vs. md: 11.0 (95% CI: 17.0 to 33.2); *P *< 0.05), but not necessarily in the CICU (*P *= 106).The most prevalent adverse events in readmitted patients were pressure ulcers (*n *= 16 (4.1%)), drug administration error (*n *= 13 (3.3%)) and enteral feeding tube (*n *= 10 (2.6%)). Meanwhile, among the nonreadmitted, phlebitis due to peripheral vein access and pressure ulcers (*n *= 42 (1.9%)), drug administration error (*n *= 41 (1.9%)) and enteral feeding tube (*n *= 31 (1.4%)). There was a tendency (*P *= 0.71) that readmitted patients were presented with higher prevalence of pressure ulcers (*n *= 16 (4.1%) vs. *n *= 42 (1.9%)).

## Conclusion

There is a higher prevalence of adverse events in readmitted patients with similarity in the type of adverse effects despite readmission.
